# Preparation of Magnetic Fe_3_O_4_/MIL-88A Nanocomposite and Its Adsorption Properties for Bromophenol Blue Dye in Aqueous Solution

**DOI:** 10.3390/nano9010051

**Published:** 2019-01-02

**Authors:** Yi Liu, Yumin Huang, Aiping Xiao, Huajiao Qiu, Liangliang Liu

**Affiliations:** Institute of Bast Fiber Crops, Chinese Academy of Agricultural Sciences, Changsha 410205, China; lyi.keai@yahoo.com (Y.L.); YuminHuang123@gmail.com (Y.H.); aipingxiao@yahoo.com (A.X.)

**Keywords:** adsorption, bromophenol blue, magnetic nanoparticles, metal-organic frameworks, wastewater

## Abstract

Metal-organic frameworks (MOFs) are considered as good materials for the adsorption of many environmental pollutants. In this study, magnetic Fe_3_O_4_/MIL-88A composite was prepared by modification of MIL-88A with magnetic nanoparticles using the coprecipitation method. The structures and magnetic property of magnetic Fe_3_O_4_/MIL-88A composite were characterized and the adsorption behavior and mechanism for Bromophenol Blue (BPB) were evaluated. The results showed that magnetic Fe_3_O_4_/MIL-88A composite maintained a hexagonal rod-like structure and has good magnetic responsibility for magnetic separation (the maximum saturation magnetization was 49.8 emu/g). Moreover, the maximum adsorption amount of Fe_3_O_4_/MIL-88A composite for BPB was 167.2 mg/g and could maintain 94% of the initial adsorption amount after five cycles. The pseudo-second order kinetics and Langmuir isotherm models mostly fitted to the adsorption for BPB suggesting that chemisorption is the rate-limiting step for this monomolecular-layer adsorption. The adsorption capacity for another eight dyes (Bromocresol Green, Brilliant Green, Brilliant Crocein, Amaranth, Fuchsin Basic, Safranine T, Malachite Green and Methyl Red) were also conducted and the magnetic Fe_3_O_4_/MIL-88A composite showed good adsorption for dyes with sulfonyl groups. In conclusion, magnetic Fe_3_O_4_/MIL-88A composite could be a promising adsorbent and shows great potential for the removal of anionic dyes containing sulfonyl groups.

## 1. Introduction

The textile industry is one of the most chemically intensive industries on Earth and the major polluter of potable water. During various stages of textile processing, huge quantities of dyes are generated in the form of wastewater [1]. Dyes usually have complex aromatic molecular structures which make them more stable and more difficult to biodegrade. As the diversity of textile products increases, different dyestuffs with highly varying chemical characteristics are used in industry, complicating further treatments of textile wastewater [2]. The direct discharge of colored and toxic wastewater into the environment affects its ecological status by causing various undesirable changes [3]. Sulfonated azo dyes, one of the aromatic sulfonates, can be easily found in the textile industry. Due to a high mobility within the aquatic system, they can easily pass through the water treatment process and cause pollution of surface water [4]. There is an urgent need for the development of effective processes to remove the dyes from wastewater.

Various physicochemical and biological methods for treating dye effluents have been reported, such as adsorption, precipitation, chemical degradation, advanced oxidation processes, biodegradation and chemical coagulation [5]. Although these methods have been widely applied, they have some disadvantages. Owing to the undesired reactions in treated water, chemical coagulation causes large amounts of sludge and extra pollution [6]. As for biological methods, it is inadequate for most textile wastewaters because of highly structured polymers with low biodegradability [7]. In the past decade, the removal of dye from aqueous solutions via adsorption has attracted much attention because of economic feasibility, simplicity, and high efficiency [8]. Traditional absorbents have some limitations such as low adsorption capacity and difficulty in separation of absorbents after reaction. Hence, it is necessary to design low cost and high-efficiency absorbents that can also be easily separated from the contaminated media [9,10]. Graphene oxide [11], metal oxides nanoparticles [12], agricultural waste peels [13], bionanomaterials [11], metal-organic frameworks (MOFs) and many kinds of materials with various modifications constantly attract researchers’ attention [14].

MOFs are a class of crystalline materials made by linking metal clusters or ions and organic linkers through covalent bonds. Owing to their highly ordered structures, high porosity and large surface areas, MOFs have attracted intensive attention in gas storage [15], molecular sensing [16], catalysis [17], energy [18], and water remediation [14]. Recently, many kinds of MOF-based materials such as rod-like metal-organic framework nanomaterial and MOF composites have been successfully synthesized and are widely used to remove dyes from wastewater [19,20,21,22]. Magnetic materials gained immense attention as adsorbents as well due to their strong magnetic response, low cost and good biocompatibility represented by ferroferric oxide (Fe_3_O_4_) nanoparticles. Fe_3_O_4_ nanoparticles could be easily separated from reaction liquids by the use of an external magnetic field. Consequently, they were widely applied in separation, catalysis and environmental remediation. The combination of Fe_3_O_4_ nanoparticles and other nanomaterials could apparently simplify procedures, save time, and improve efficiency in adsorption and separation fields [23].

Bromophenol blue (BPB) and its structurally related derivatives have been extensively applied in many industries like food, cosmetic, textile, printing inks and laboratory indicators [24]. The present study reports the successful synthesis of magnetic composite Fe_3_O_4_/MIL-88A and its use for the adsorption of BPB in order to evaluate its feasibility as a novel adsorbent in environmental remediation. MIL-88A was a 3D structured framework built up from trimers of Fe^3+^ octahedra linked to fumarate dianions. This structure exhibited a pore-channel system along the c axis and cages (5–7 Å) [25]. In addition, MIL-88A exhibited a flexible framework and possessed active iron metal sites, which were applied as a photocatalyst [26]. The synthesized Fe_3_O_4_/MIL-88A composite was characterized with transmission electron microscopy (TEM), scanning electron microscopy (SEM), X-ray powder diffraction (XRD), thermogravimetric analysis (TGA) and vibration sample magnetometer (VSM). The adsorption properties for BPB were investigated in terms of the effects of contact time, adsorbent dosage and initial dye concentration on removal efficiency of BPB and the kinetic and isotherm of adsorption process. As a superior adsorbent material, Fe_3_O_4_/MIL-88A showed proper magnetic response for shortening reaction time and excellent adsorption ability for the removal of dyes.

## 2. Materials and Methods

### 2.1. Materials

The chemicals, sodium acetate, fumaric acid, ethylene glycol, ferric chloride, ethanol, ferrous sulfate and ammonium hydroxide were of analytical grade and obtained from Sigma-Aldrich Chemicals (St. Louis, MO, USA). The dyes, Bromophenol Blue (BPB), Bromocresol Green, Brilliant Green, Brilliant Crocein, Amaranth, Fuchsin Basic, Safranine T, Malachite Green and Methyl Red were obtained commercially from Sinopharm Chemical Reagent Co., Ltd. (Shanghai, China). Ultrapure water (18.2 MΩ cm resistivity) was obtained from an ELGA water purification system (ELGA Berkefeld, Veolia, Germany). All other chemicals were also analytical grade and purchased from Sinopharm Chemical Reagent Co., Ltd. (Shanghai, China).

### 2.2. Synthesis of Magnetic Fe_3_O_4_/MIL-88A Composite

The MIL-88A was prepared according to the previous synthesis customs with some modifications in the solution concentration and reaction time [26]. Typically, 10 mmol of FeCl_3_·6H_2_O and 10 mmol of fumaric acid were first dissolved in 25 mL of water, and then the homogeneous solution was transferred into a 120 mL Teflon-lined stainless steel autoclave and heated to 65 °C for 12 h. After cooling to room temperature, the product was dispersed in water under ultrasonic waves for several minutes and centrifuged. The liquid supernatant was decanted and the precipitate (the weight was 0.89 g after drying) was re-dispersed in 100 mL of water for further use.

The magnetic Fe_3_O_4_/MIL-88A composite was prepared by coprecipitation method [27]. 3 mmoL of FeCl_3_·6H_2_O and 1.5 mmoL of FeSO_4_·7H_2_O were mixed in 200 mL of water to form an aqueous solution. The solution was transferred into a round bottom flask containing 100 mL of MIL-88A aqueous solution under mechanical stirring in water bath at 75 °C. While mechanical stirring, 3 mL of ammonium hydroxide was added dropwise into the flask and the color of the solution became black indicating precipitate formation. The mixture was vigorously stirred for 30 min at 75 °C and this continued for 90 min at room temperature. After the reaction, the Fe_3_O_4_/MIL-88A composite was magnetically separated using a magnet and washed with water and ethanol three times. Finally, it was dried in a vacuum oven at 45 °C for 12 h (1.20 g after drying).

### 2.3. Characterizations

In order to confirm the morphology and structure of the final products, the synthesized magnetic Fe_3_O_4_/MIL-88A composites were characterized by means of TEM, field emission scanning electron microscopy (FESEM), and XRD. Specifically, TEM images of magnetic Fe_3_O_4_/MIL-88A composites were recorded on a Tecnai-G20 transmission electron microscope (FEI, Hillsboro, OR, USA). FESEM images were recorded on a JSM-7500F Field Emission Scanning Electron Microscope (JEOL, Tokyo, Japan). The XRD spectra were recorded using a powder X-ray Diffractometer (Rigaku RINT 2500, Rigaku Corporation, Tokyo, Japan) with Cu/Kα radiation at 30 mA and 40 kV. TGA was performed in nitrogen atmosphere from 40 to 800 °C with a heating rate of 10 °C/min with a simultaneous thermal analyzer (Netzsch STA 449F3, Ahlden, Germany). Moreover, the magnetic properties of Fe_3_O_4_/MIL-88A composites were measured at room temperature on a vibration sample magnetometer VSM7407 (Lake Shore, Westerville, OH, USA).

### 2.4. Adsorption Experiments

The adsorption rate experiments were performed by immersing 0.2 g of Fe_3_O_4_/MIL-88A powder into 50 mL of 1.2 mg/mL of dye aqueous solutions in a 100 mL conical flask with cover. The flask was shaken using a mechanical shaker (SHA-CA, Changzhou, China) at 27 °C and 200 rpm for 135 min. At each period of time, about 2.0 mL of the solution was picked up and filtrated through a syringe filter to measure the concentration of BPB using an ultraviolet-visible (UV-Vis) spectrophotometer (UV-2700, Shimadzu, Kyoto, Japan) at a wavelength of 590 nm. Different process variables such as initial concentration (0.3–1.5 mg/mL) and doses (0.05–0.4 g) were also investigated. Percentage removal of dyes was determined using the following equation [28]:

(1) $$\mathrm{Removal}\;\mathrm{efficiency}\;(\%)=\frac{\left(C_{0}-C_{t}\right)}{C_{0}}\times 100\%$$

where *C*_0_ represents the initial concentration of dye and *C_t_* represents the concentration of dye after *t* minutes. The equilibrium amount of adsorption (*q_e_*) and the amount of adsorption (*q_t_*) at given time were calculated according to the following equation [29]:

(2) $$q_{e}=\frac{(C_{0}-C_{e})\times V}{W}$$

(3) $$q_{t}=\frac{(C_{0}-C_{t})\times V}{W}$$

where *C_e_* is the equilibrium concentration of dye (mg/mL), *V* is the solution volume (mL), and *W* is the adsorbent mass (g).

## 3. Results and Discussion

### 3.1. Characterization of Magnetic Fe_3_O_4_/MIL-88A Composite

#### 3.1.1. Transmission Electron Microscopy (TEM) and Field Emission Scanning Electron Microscopy (FESEM)

**Figure 1:**
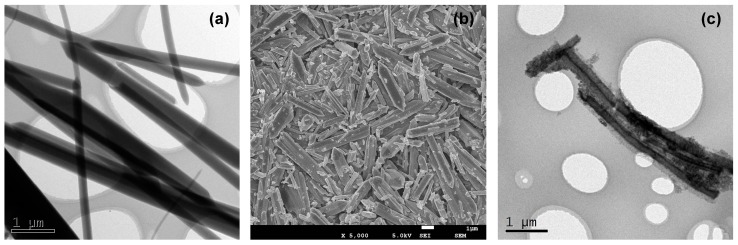
The transmission electron microscopy (TEM) (**a**) and field emission scanning electron microscopy (FESEM) (**b**) images of MIL-88A and the TEM (**c**) image of magnetic Fe_3_O_4_/MIL-88A composite.

The MIL-88A and magnetic Fe_3_O_4_/MIL-88A composite were characterized by TEM and FESEM to visually observe the morphologies changes during synthesis processes. TEM image (Figure 1a) showed the prepared MIL-88A were crystallized hexagonal microrods of over 5 μm in length and about 500 nm in diameter. FESEM observation (Figure 1b) confirmed the microrod shape and revealed that the size distribution of these MIL-88A was relatively uniform with some exceptions. After the combination of Fe_3_O_4_ nanoparticles, the TEM image (Figure 1c) showed many Fe_3_O_4_ nanoparticles were grown on the surface of MIL-88A and the structure of MIL-88A was retained. The diameter of Fe_3_O_4_ nanoparticles were about 5 to 10 nm. It could be seen that the magnetic Fe_3_O_4_/MIL-88A composite was successfully prepared and showed characteristics of both Fe_3_O_4_ nanoparticles and MIL-88A in nanostructure.

#### 3.1.2. X-Ray Powder Diffraction (XRD)

**Figure 2:**
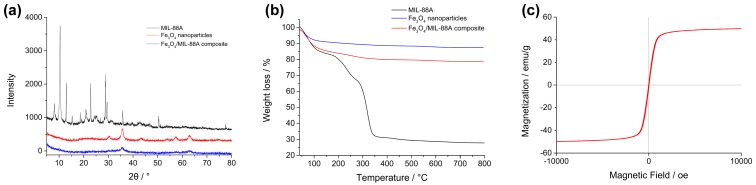
(**a**) The X-ray powder diffraction (XRD) patterns of MIL-88A (black), Fe_3_O_4_ nanoparticles (red) and magnetic Fe_3_O_4_/MIL-88A composite (blue); (**b**) thermogravimetric analysis (TGA) curves of MIL-88A (black), Fe_3_O_4_ nanoparticles (blue) and magnetic Fe_3_O_4_/MIL-88A composite (red); (**c**) The magnetization curve of magnetic Fe_3_O_4_/MIL-88A composite.

The structures of MIL-88A and Fe_3_O_4_/MIL-88A composite were analyzed by XRD and the spectra were compared with that of Fe_3_O_4_ nanoparticles. As shown in Figure 2a, the spectrum of MIL-88A showed peaks at 8.14°, 10.42° and 12.98°, which was accordance with the reported information [30]. Meanwhile, the spectrum of Fe_3_O_4_ nanoparticles also showed characteristic peaks at 30.48°, 35.72°, 43.32°, 57.56° and 62.86° corresponding the indices (220), (311), (400), (511) and (440). This pattern was in agreement with previously reported Fe_3_O_4_ crystal XRD data [31]. Finally, the spectrum of magnetic Fe_3_O_4_/MIL-88A composite exhibited some characteristic peaks of Fe_3_O_4_ nanoparticles at 35.72° and 62.86°. However, the peaks of MIL-88A were almost missed. As shown by TEM, the MIL-88A was coated with Fe_3_O_4_ nanoparticles, which might interfere the diffraction peak of MIL-88A crystals. Moreover, the quantity of Fe_3_O_4_ nanoparticles were much more than MIL-88A, therefore the diffraction signals of Fe_3_O_4_ nanoparticles were much higher than those of MIL-88A and masked the signals of MIL-88A.

#### 3.1.3. Thermogravimetric Analysis (TGA)

Figure 2b shows the weight losses of MIL-88A, Fe_3_O_4_ nanoparticles and magnetic Fe_3_O_4_/MIL-88A composite. In nitrogen below 350 °C, the weight loss of MIL-88A was attributed to the collapse of organic skeleton [32]. The weight loss of Fe_3_O_4_ nanoparticles below 100 °C was related to the evaporation of absorbed water, while the weight loss above 100 °C was relatively flat without obvious change. Finally, magnetic Fe_3_O_4_/MIL-88A composite showed the same tendency in weight loss as that of MIL-88A and the final weight loss was in between the former two materials. It illustrated that the combination of MIL-88A and Fe_3_O_4_ nanoparticles was effective.

#### 3.1.4. Vibration Sample Magnetometer (VSM)

As a kind of magnetic nanomaterials, the magnetic property of magnetic Fe_3_O_4_/MIL-88A composite was evaluated by VSM as well. The magnetization curves of magnetic Fe_3_O_4_/MIL-88A composite was shown in Figure 2c. It could be found that the maximum saturation magnetization reached 49.8 emu/g. This value was less than that of bare Fe_3_O_4_ (about 65.0 emu/g) due to the existence of MOF without magnetic response. However, the prepared Fe_3_O_4_/MIL-88A composite was sufficient for magnetic separation in experiments and could be separated from solution within two minutes.

#### 3.1.5. Adsorption Ability

The adsorption ability of magnetic Fe_3_O_4_/MIL-88A composite was verified and compared with that of MIL-88A and Fe_3_O_4_ nanoparticles. Under the same adsorption conditions, the adsorption amount of magnetic Fe_3_O_4_/MIL-88A composite was 141.5 mg/g with removal efficiency of 26.5%. While, the adsorption amounts of MIL-88A and Fe_3_O_4_ nanoparticles were 140.6 mg/g and 13.6 mg/g, respectively. It could be seen that MIL-88A and magnetic Fe_3_O_4_/MIL-88A composite had considerable adsorption abilities for BPB. However, the adsorption ability of Fe_3_O_4_ nanoparticles rather poor. The combination of two kinds of materials gave the magnetic responsibility to MIL-88A and maintained the adsorption ability. Based on these, the prepare of magnetic Fe_3_O_4_/MIL-88A composite could be considered successful.

### 3.2. Effects of Parameters on Dye Adsorption

#### 3.2.1. Effect of Contact Time

**Figure 3:**
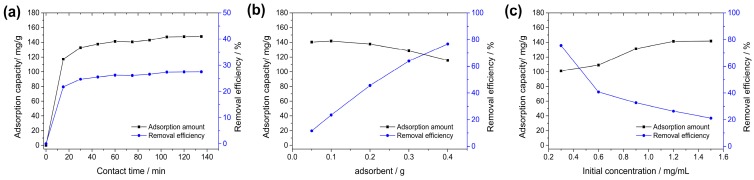
Effects of contact time (**a**), adsorbent dosage (**b**) and initial concentration (**c**) on the adsorption amount of Fe_3_O_4_/MIL-88A composite (black) and removal efficiency (blue).

The effect of contact time (15–135 min) on the removal efficiency of BPB was shown in Figure 3a. At the initial stage of adsorption, an increasing adsorption could be observed. However, the increase of adsorption slowed down as the adsorption proceeded, and finally the adsorption reached saturation. The maximum adsorption amount (141.2 mg/g) was achieved at 60 min with removal efficiency of 26.2%. After 135 min of adsorption, no significant increase in adsorption amount was observed (141.2 mg/g to 147.9 mg/g). Similar patterns could be obtained in many adsorption experiments of dyes [33].

#### 3.2.2. Effect of Adsorbent Dosage

The effect of adsorbent dosage was investigated by addition of various amounts of magnetic Fe_3_O_4_/MIL-88A composite in 50 mL of dyes solution (1.2 mg/L) at room temperature for 60 min. As shown in Figure 3b, the adsorption amount of Fe_3_O_4_/MIL-88A composite decreased and removal efficiency increased with increasing dosage. The removal efficiency of BPB increased from 11.6% to 76.7%, which might due to the increase of adsorption sites on Fe_3_O_4_/MIL-88A surface were available for adsorption to dyes [34]. However, the adsorption capacity of Fe_3_O_4_/MIL-88A composite of decreased from 140.2 mg/g to 115.6 mg/g and showed maximum of 141.7 mg/g at 0.1 g. This kind of trend was commonly shown in many adsorption researches, which was caused by the agglomeration of adsorbent at high dosage, resulting in the reduction of effective sites on the adsorbent surface [35].

#### 3.2.3. Effects of Initial Dye Concentration

The initial concentrations have great influences in this system, because it provides driving force to overcome mass transfer resistance between dye ion and solid phase [36]. The effect of initial dye concentrations (0.3–1.5 mg/mL) on the adsorption on magnetic Fe_3_O_4_/MIL-88A composite was shown in Figure 3c. It could be found that the removal efficiency of BPB decreased from 75.6% to 21.2% accompanied by the increase of adsorption capacity of Fe_3_O_4_/MIL-88A composite (101.2 mg/g to 141.7 mg/g) with initial dye concentration increased. The increase of adsorption capacity of absorbent could also be observed in the adsorption of R-250 dye on starch/poly(alginic acid-*cl*-acrylamide), direct orange 34 on natural clay and crystal violet on polyaniline nanoparticles [36,37,38]. However, the reduction of removal efficiency with increasing initial dye concentration might be attributed to relatively limited number of active sites for dyes compared with the increasing dye molecules.

### 3.3. Adsorption Kinetics

In order to study the mechanism of adsorption kinetics, two kinds of commonly used kinetic models, pseudo-first-order and pseudo-second-order, were applied in this research to study the adsorption behavior of BPB on magnetic Fe_3_O_4_/MIL-88A composite. Especially, the pseudo-first-order model was the simplest model and widely exploited for investigating the adsorption behavior. The pseudo-first-order model was expressed as follows [39]:

(4) $$\ln\;(q_{e}-q_{t})=\ln\;q_{e}-k_{1}t$$

where *q_t_* (mg/g) and *q_e_* (mg/g) are the amounts of adsorbed dyes at a certain time and at equilibrium status respectively, *t* is contact time (min) and *k*_1_ (min^−1^) is the pseudo-first order rate constant. The pseudo-second-order model could be represented as:

(5) $$\frac{t}{q_{t}}=\frac{1}{k_{ad}q_{e}^{2}}+\frac{1}{q_{e}}t$$

where *k_ad_* (g/mg/min) is the pseudo-second-order rate constant.

**Figure 4:**
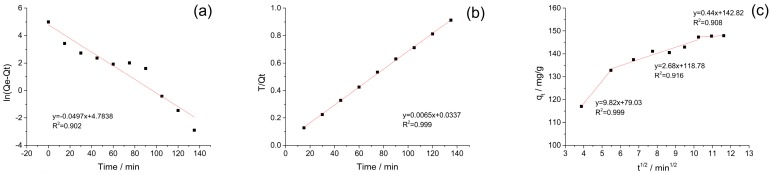
Adsorption kinetics plots (black dot) and the fitting curve (line) for the adsorption of Bromophenol Blue (BPB) on magnetic Fe_3_O_4_/MIL-88A composite at 27 °C: pseudo-first-order kinetic models (**a**) and pseudo-second-order kinetic models (**b**) and intraparticle diffusion model (**c**).

Figure 4 and Table 1 illustrated the linear plots of first and second order models for the adsorption of BPB on magnetic Fe_3_O_4_/MIL-88A composite. To estimate the suitability of two models, the corresponding correlation coefficients (*R*^2^) were obtained by linear regression methods and higher *R*^2^ value indicated more applicable model for describing the kinetics of BPB adsorption. As a result, the higher *R*^2^ value for pseudo-second-order model (0.999) indicated this model was in good agreement with the experimental values and was more suitable for this adsorption. However, the *R*^2^ for pseudo-first-order (0.902) is much lower than that offered by the pseudo-second-order model, which indicated the pseudo-first-order model was not suitable for the adsorption of BPB. This result reflected the rate limiting step for this adsorption might be chemisorption, involving valence force via sharing or exchanging electron between adsorbent and adsorbate [40].

In order to determine the adsorption process mechanism, an intraparticle diffusion model was also used to determine the rate-limiting step during the adsorption process [41]. The expression of this model is shown as the following equation:

(6) $$q_{t}=k_{i}t^{0.5}+C$$

where *C* (mg/g) is the intercept in intraparticle diffusion plot, and *k_i_* (mg/g/min) is the intraparticle diffusion rate constant. Figure 4c showed linear plots in three sections, implying that three steps were involved in the adsorption with decreasing rates: (a) surface adsorption; (b) intraparticle diffusion; (c) adsorption close to equilibrium [42]. The rate constants *k_i_* decreased and *C* values increased from step (a) to step (c) showed the increased contribution of the boundary layer to the adsorption rate. This kind of evolution was reported in other dye/adsorbent systems [43].

### 3.4. Adsorption Isotherms

The analysis on adsorption equilibrium could reveal types of adsorbate layers formed on the adsorbent surface. Three isotherm models were used in this study including the Langmuir model, Freundlich model and Temkin model. The Langmuir model assumed that uptake occurs on a homogeneous surface by monolayer adsorption without interaction between the absorbed materials, which could be expressed as following [44]:

(7) $$\frac{C_{e}}{q_{e}}=\frac{1}{bq_{m}}+\frac{C_{e}}{q_{m}}$$

where *C_e_* (mg/mL) and *q_e_* (mg/g) represent the equilibrium concentration of dye and adsorption capacity at equilibrium, *q_m_* (mg/g) represents the maximum adsorption capacity and *b* represents the equilibrium adsorption constant (mL/mg).

The Freundlich model described the formation of multilayers by adsorbate molecules on the adsorbent surface because of different affinities for various active sites on adsorbent surface [45]. The equation was expressed as following:

(8) $$\log\;q_{e}=\log\;k+\frac{1}{n}\log\;C_{e}$$

where *k* (mL/mg) and *n* are the Freundlich constants.

The Temkin model assumed that adsorbent-adsorbate interactions could not be neglected during the adsorption mechanism, and the heat of adsorption decreases linearly with the adsorbate coverage due to the interaction [46]. This model could be represented by the following equation:

(9) $$q_{e}=B\log\;A_{T}+B\log\;C_{e}$$

where *B* (mg/g) and *A_T_* (mL/mg) are the Temkin isotherm equilibrium binding constant.

**Figure 5:**
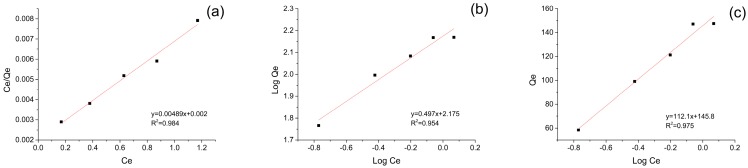
Adsorption isotherms plots (Black dot) and the fitting curve (Red line) for the adsorption of BPB dye on magnetic Fe_3_O_4_/MIL-88A composite at 27 °C: Langmuir model (**a**), Freundlich model (**b**) and Temkin model (**c**).

The experimental data were fitted to the Langmuir, Freundlich and Temkin models as described in Figure 5 and the detail parameters were shown in Table 2. Through the comparison on the *R*^2^ values (0.984 for Langmuir, 0.954 for Freundlich and 0.975 for Temkin, respectively), the Langmuir model appeared to be the most suitable model in describing adsorption of BPB on magnetic Fe_3_O_4_/MIL-88A composite. Thus, the adsorption of BPB was typical monomolecular-layer adsorption.

### 3.5. Comparison Study

**Figure 6:**
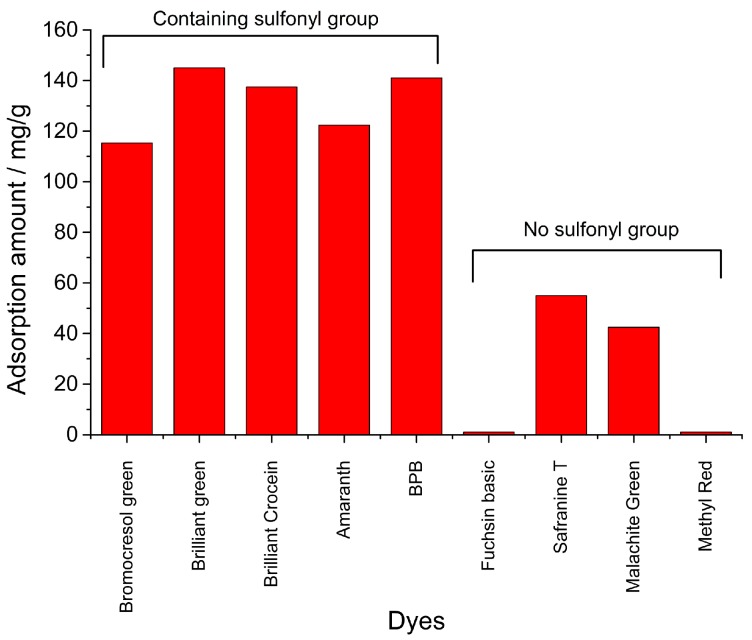
Comparison of the adsorption amount among various dyes on magnetic Fe_3_O_4_/MIL-88A composite.

Eight dyes, Bromocresol Green, Brilliant Green, Brilliant Crocein, Amaranth, Fuchsin Basic, Safranine T, Malachite Green and Methyl Red, were investigated and compared at the same adsorption conditions (Figure 6). 0.1 g of Fe_3_O_4_/MIL-88A powders were transferred into 50 mL of 1.0 mg/mL of dye solutions, the mixture was shaken at 27 °C for 60 min. The adsorption amount was calculated through monitoring the change of absorbance for each dye solution. Among these dyes, Bromocresol Green, Brilliant Green, Brilliant Crocein, Amaranth, and BPB all contain sulfonyl groups. However, there is no sulfonyl group in the structure of the other four dyes, Fuchsin Basic, Safranine T, Malachite Green and Methyl Red. As a result, five dyes containing sulfonyl groups showed much better adsorption amounts than that of dyes without sulfonyl group. In particular, there was nearly no adsorption for Fuchsin Basic and Methyl Red. Some computed and experimental properties were listed in Table 3. Topological polar surface area (TPSA) values were obtained on the Pubchem website (https://pubchem.ncbi.nlm.nih.gov/compound/) and pKa values were obtained on the Chemicalbook website (https://www.chemicalbook.com/). The TPSA was defined as the sum of surfaces of polar atoms in a molecule. This property has been shown to correlate with the human intestinal absorption and blood–brain barrier penetration. Herein, BPB, Bromocresol Green, Brilliant Green, Brilliant Crocein and Amaranth with higher adsorption amounts showed relative higher TPSA values and lower pKa values. Based on these results, it might be assumed that magnetic Fe_3_O_4_/MIL-88A composite could effectively adsorbed dyes with sulfonyl groups, and the polar surface and pKa values of molecules might affect the adsorption. However, the detailed mechanism, especially in the surface charge of absorbent and dyes, still needs more in-depth and systematic studies in future [47].

Adsorption capacities of various adsorbents for BPB as reported in literature were presented in Table 4. The comparison between this work and other reported data showed that magnetic Fe_3_O_4_/MIL-88A composite was a satisfied adsorbent for BPB compared to other adsorbents, as the adsorption capacity of magnetic Fe_3_O_4_/MIL-88A composite was higher than that of most reported materials. Therefore, it could be safely concluded that the materials prepared in this work exhibited considerable ability for adsorbing BPB from aqueous solutions.

### 3.6. Recycling of Fe_3_O_4_/MIL-88A Composite

**Figure 7:**
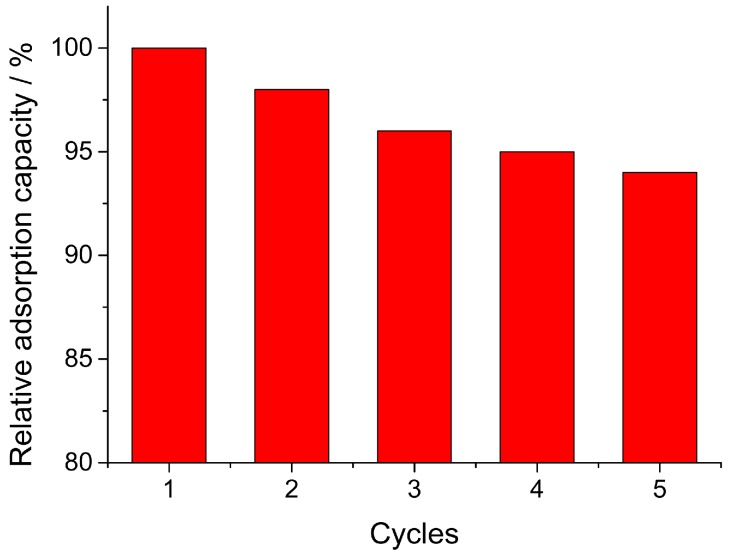
Reusability of magnetic Fe3O4/MIL-88A composite.

The reuse of adsorbent is an important aspect for practical application in economic aspect. To evaluate the reusability of magnetic Fe_3_O_4_/MIL-88A composite, the adsorbed composite was desorbed with ethanol solution and used for next adsorption cycles. The adsorption capacity of each cycle was monitored and the relative adsorption capacity was calculated by comparing with the first run in percentage form (adsorption capacity defined as 100%). Five cycles’ reuse of magnetic Fe_3_O_4_/MIL-88A composite was shown in Figure 7. It could be seen that Fe_3_O_4_/MIL-88A maintained high adsorption capacity (94%) without significant loss after five cycles. The result demonstrated that Fe_3_O_4_/MIL-88A composite could be applied in practical application owing to their high adsorption capacity and good reusability.

## 4. Conclusions

The magnetic Fe_3_O_4_/MIL-88A composite was prepared and characterized by TEM, FESEM, XRD, TGA and VSM. The characterizations showed the preparation was successful and sufficient for magnetic separation. The magnetic Fe_3_O_4_/MIL-88A composite showed good adsorption ability for BPB and other dyes containing sulfonyl groups. The adsorption amount of magnetic Fe_3_O_4_/MIL-88A composite was higher than many reported materials for BPB and could be maintained during five cycles. The results illustrated that the magnetic Fe_3_O_4_/MIL-88A composite has promising application in dye-contaminated wastewater treatment, especially for anionic dyes containing sulfonyl groups.

## Figures and Tables

**Table 1:** Adsorption kinetic parameters for BPB on magnetic Fe_3_O_4_/MIL-88A composite.

Exp	Pseudo-First-Order Model			Pseudo-Second-Order Model			Intraparticle Diffusion		
*q_exp_*	*q_e_*	*k*_1_	*R* ^2^	*q_e_*	*k_ad_*	*R* ^2^	*k*_1_	*k*_2_	*k*_3_
148.0	119.56	0.0497	0.902	153.85	0.00125	0.999	9.82	2.68	0.44

**Table 2:** The adsorption isotherm parameters for BPB on magnetic Fe_3_O_4_/MIL-88A composite.

Langmuir			Freundlich			Temkin		
*q_m_*	*b*	*R* ^2^	1/*n*	*k*	*R* ^2^	*B*	*A_T_*	*R* ^2^
204.50	2.445	0.984	0.497	149.62	0.954	112.1	19.98	0.975

**Table 3:** The chemical properties of investigated dyes.

Dyes	TPSA/A^2^	pKa
BPB	92.2	3.85
Bromocresol Green	92.2	4.7
Brilliant Green	92.1	2.5
Brilliant Crocein	197	- ^a^
Amaranth	238	-
Fuchsin Basic	75.9	-
Safranine T	68.8	6.4
Malachite Green	6.2	6.9
Methyl Red	65.3	4.95

^a^ means the value could not be found in the website.

**Table 4:** A comparison of adsorption of BPB by various reported adsorbents.

Adsorbent	Adsorbate	Adsorption Capacity (mg/g)	Reference
α-Chitin nanoparticles	BPB	22.72	[48]
Activated charcoal	BPB	About 9.0 × 10^−3^	[49]
SiO_2_·Bth^+^·PF_6_^−^ ionic liquids	BPB	238.10	[24]
Modified layered silicate	BPB	184.5	[50]
Sorel’s cement nanoparticles	BPB	4.88	[51]
Polymer-clay composite	BPB	About 7.5	[52]
Mesoporous MgO nanoparticles	BPB	40	[53]
Mesoporous hybrid gel	BPB	18.43	[54]
CoFe_2_O_4_ nano-hollow spheres	BPB	29.3	[55]
Graphene oxide functionalized magnetic chitosan composite	BPB	9.5	[56]
CuS-NP-AC	BPB	106.4	[57]
Fe_2_O_3_-ZnO-ZnFe_2_O_4_/carbon nanocomposite	BPB	90.91	[58]
Iron oxide nanoparticles	BPB	About 110	[59]
Fe_3_O_4_/MIL-88A	BPB	141.9–167.2	This study
